# Correction: Populations of arable weed species show intra-specific variability in germination base temperature but not in early growth rate

**DOI:** 10.1371/journal.pone.0307861

**Published:** 2024-07-23

**Authors:** Jana Bürger, Andrey V. Malyshev, Nathalie Colbach

Fig 1 was plotted with a censored subset of the original climate data. Please see the correct Fig 1 here.

**Fig 1:**
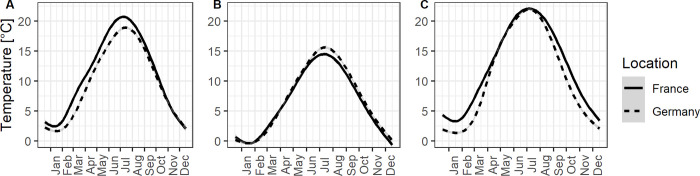
Average daily temperatures in France (Bretenières near Dijon) and Germany (Warnemünde near Rostock) between 2000 and 2019. A. Mean air temperature. B. Minimum air temperature. C. Mean soil temperature (10cm below ground). GAM smoothing over daily values, grey area is 95% confidence interval. Weather stations at the INRA experimental station of Dijon-Epoisses, and German Weather Service (DWD) Rostock-Warnemünde (Datasources: platform INRA CLIMATIK, www.opendata.dwd.de).
