# Supplementary material for: Populations of arable weed species show intra-specific variability in germination base temperature but not in early growth rate
Source: PLoS One. 2020 Oct 9;15(10):e0240538. doi: 10.1371/journal.pone.0240538 (PMC7546504; doi:10.1371/journal.pone.0240538)
Supplement: S1 File — (DOCX) [file pone.0240538.s001.docx]

Content

[Supporting information 1](#_Toc48658842)

[1. Seed sources 1](#_Toc48658843)

[2. Determining the germination base temperature – graphic example 2](#_Toc48658844)

[3. Determining the end of exponential growth period – graphic example 3](#_Toc48658845)

[4. Experiments included in the analyses of this manuscript either in main part or in supporting information 3](#_Toc48658846)

[5. Intra- and inter-specific variability of relative growth rate 4](#_Toc48658847)

[5.1. Effect of experimental location 4](#_Toc48658848)

[5.2. Effect of experimental conditions on early relative growth rate 5](#_Toc48658849)

[5.3. Effects of population and species as reported in the manuscript 5](#_Toc48658850)

[5.4. Effects of population and species: analysis repeated including seeds tested at their non-native location 6](#_Toc48658851)

[6. Pairwise comparison of means between populations of the same species 8](#_Toc48658852)

[7. Estimates of early relative growth rate per species 9](#_Toc48658853)

[7.1. Model used to estimate species means 9](#_Toc48658854)

[7.2. Estimates 9](#_Toc48658855)

[References 10](#_Toc48658856)

## Seed sources

Table 1: Seed sources for experiments on germination base temperature and relative growth rate in Rostock, 2016 and 2017. Abbreviations: EG – experimental garden University Rostock, ES – experimental station Stover Acker, CS – commercial supplier, INRA – seed collection INRA Dijon. SP – spring, SU – summer, AU – autumn.

| Species | EPPO Code | Seed source and harvest date - German provenance | Temperature levels (°C) of base temperature experiment a | Seed source and harvest date - French provenance used in Rostock |
| --- | --- | --- | --- | --- |
| *Alopecurus myosuroides* | ALOMY | EG 07/2016 | 7.1; 11; 15; 16.3 | INRA pop. ID unknown |
| *Amaranthus retroflexus* | AMARE | ES 09/2015 | 20; 22; 24; 26 | INRA pop. ID unknown |
| *Anchusa arvensis* | LYCAR | EG 2016 | 19; 21; 23; 25 |  |
| *Apera spica-venti* | APESV | ES 08/2015 | 7.1; 11; 16.3 |  |
| *Capsella bursa-pastoris* | CAPBP | ES 06/2014 | - |  |
| *Centaurea arvensis* | CENCY | EG 06/2016, CS 2016 | 7.1; 11; 16.3 |  |
| *Chenopodium album* | CHEAL | ES 2015 | - | INRA pop. ID unknown |
| *Digitaria sanguinalis* | DIGSA | ES 2011 | - |  |
| *Echinochloa crus-galli* | ECHCG | ES 08/2015 | 11; 13.9; 16.3; 18.4 | INRA pop.984 Herbiseed |
| *Galium aparine* | GALAP | EG 07/2016 | - |  |
| *Geranium dissectum* | GERDI | CS 2016 | 6; 7.1; 10.1; 11; 13.9 |  |
| *Matricaria chamomilla* | MATCH | ES 8/2015 | 11; 13.9; 16.3; 18.4 |  |
| *Matricaria inodora* | MATIN | ES 2015 | 6; 8.6; 13.9; 18.4 |  |
| *Papaver rhoeas* | PAPRH | EG 07/2016 | 6; 8.6; 12.1; 13.9 |  |
| *Poa annua* | POAAN | EG 2013 | - |  |
| *Setaria viridis* | SETVI | EG 2011 | 15; 19.9; 21.9; 23.7 | INRA pop.366 Burgundy |
| *Solanum nigrum* | SOLNI | ES 2011 | - | INRA pop.1006 Herbiseed |
| *Sonchus asper* | SONAS | ES 2016 | - |  |
| *Stellaria media* | STEME | EG 06/2016 | 6; 7.1; 8.6; 11; 13.9; 16.3 |  |
| *Sysimbrium officinale* | SYSOF | EG 9/2016 | 7.1; 12.1; 15 |  |
| *Veronica hederifolia* | VERHE | EG 2008 | - |  |
| *Viola arvensis* | VIOAR | ES 06/2016 | - |  |

## Determining the germination base temperature – graphic example

Figure 1: Determination of germination base temperature using Stellaria media as an example. A. Germination curves for different constant temperatures fitted to germination observations vs time in days (n=4 per temperature level). B. Linear regression of germination rate on temperature. Germination base temperature is determined as the intercept with the x-axis (2.3°C ± 0.2 for S. media).

## Determining the end of exponential growth period – graphic example

Figure 2: Principle for determining the end of the initial exponential growth period. A. A polynomial regression ax²+bx+c was fitted to all observations corresponding to a pot (here pot 2 for Zea mays in 2012). The slope at any point x is the derivate 2ax+b. The sThe slope at TT=0 is b. The end of the exponential growth period is fixed as TTf where the slope is b/10. B. Initial leaf area LA0 and relative growth rate RGR are estimated by fitting a linear regression y=bx+c to the data where TT<TTf, and LA0 = exp(c) and RGR=b. [1].

## Experiments included in the analyses of this manuscript either in main part or in supporting information

Table 2: Species included in experiments on germination base temperature and early growth rate. The paper reports on experimental results from Rostock, and uses additional results from earlier experiments in Dijon to analyse intra-specific variability. Meaning of symbols: ”x”: species was tested, “-“: species was not tested.

| Experiment | Base temperature | Relative growth rate | Relative growth rate | Relative growth rate |
| --- | --- | --- | --- | --- |
| Experimental location | Rostock | Rostock | Rostock | Dijon |
| Seed provenance | German | German | French | French |
| Species |  |  |  |  |
| *Alopecurus myosuroides* | x | x | x | x |
| *Amaranthus retroflexus* | - | x | x | x |
| *Anchusa arvensis* | x | x | *-* | *-* |
| *Apera spica-venti* | x | x | *-* | *-* |
| *Capsella bursa-pastoris* | - | x | *-* | x |
| *Centaurea arvensis* | x | x | *-* | x |
| *Chenopodium album* | x | x | x | x |
| *Digitaria sanguinalis* | - | x | *-* | x |
| *Echinochloa crus-galli* | x | x | x | x |
| *Galium aparine* | - | x | *-* | x |
| *Geranium dissectum* | x | x | *-* | x |
| *Matricaria chamomilla* | x | x | - | x |
| *Matricaria inodora* | x | x | - | x |
| *Papaver rhoeas* | x | x | - | - |
| *Poa annua* | - | x | - | x |
| *Setaria viridis* | x | x | x | x |
| *Solanum nigrum* | - | x | x | x |
| *Sonchus asper* | - | x | - | x |
| *Stellaria media* | x | x | - | x |
| *Sisymbrium officinale* | x | - | - | - |
| *Veronica hederifolia* | - | x | - | - |
| *Viola arvensis* | - | x | - | x |

## Intra- and inter-specific variability of relative growth rate

## Effect of experimental location

Table 3 : Effect of experimental location on species relative growth rates (RGR). Type III ANOVA of the effect of species and experiment on RGR with Satterthwaites' method estimation of degrees of freedom, Season included as random effect. Six species used. At different times. Population nested in species. Three species with interaction species: provenance.

|  | Numerator Degrees of Freedom | Denominator Degrees of Freedom | F | p |
| --- | --- | --- | --- | --- |
| Species | 5 | 36.7 | 3.16 | 0.018 * |
| Location | 1 | 7.9 | 1.03 | 0.34 |
| Species: Location | 6 | 208.4 | 8.88 | <0.001 *** |

## Effect of experimental conditions on early relative growth rate

If species were tested in more than one repetition, RGR increased by approx. 0.01 cm²/ cm²*°C*days if a repetition took place one month later.

Figure 3: Relative growth rates of six weed species during seedling stage. Measurements from two experimental series in Dijon (Dij) and Rostock (Ros). Seed provenance either from France (F) or from Germany (D). Number of plants: ALOMY: 9/9/4, AMARE: 5/10/12/4/4/4, CHEAL: 10/9/5/5/9/9, ECHCG: 2/10/7/5, SETVI: 24/9/13, SOLNI: 9/4/14/9/10.

## Effects of population and species as reported in the manuscript

Table 4: Species and population effects on early relative growth rate. Analysis of variance table of fixed effects for 16 species with two provenances (German/ French). Random effects were experimental location and temporal repetition. η²: proportion of variance accounted for by each of the main effects. Variance explained by fixed effects in the model (marginal): 0.61, variance explained by whole model (conditional): 0.79, variance explained by the grouping structure (ICC) = 0.47. Model included data of: German populations tested in Rostock and French provenances in Dijon, but NOT French provenances tested in Rostock.

|  | SSQ | MeanSQ | Numerator Degrees of Freedom | Den. Degrees of Freedom | F | p |  | η² |
| --- | --- | --- | --- | --- | --- | --- | --- | --- |
| Species | 0.007 | 0.00045 | 15 | 87.4 | 14.05 | <0.001 | *** | 0.303 |
| Population | 0.000 | 0.00000 | 1 | 9.3 | 0.01 | 0.93 |  | 0.000 |
| Species : Population | 0.003 | 0.00022 | 15 | 87.4 | 6.98 | <0.001 | *** | 0.151 |
| Residuals | 0.012 |  |  |  |  |  |  |  |

## Effects of population and species: analysis repeated including seeds tested at their non-native location

The results of our analysis varied slightly when we included the experimental data of French populations tested in Rostock into the dataset. With these plants not grown in the original environment of their mother plants, provenance had a significant, but very small effect on relative growth rate (Eta² =0.006, p=0.03) in a mixed model similar to the one presented in the main text.

Table 5: Species and population effects on early relative growth rate. Analysis of variance table of fixed effects for 16 species with two populations from German or French provenance. η²: proportion of variance accounted for by each of the main effects. ICC: Intraclass correlation coefficient - proportion of the variance explained by the grouping structure in the model (random effect). Variance explained by fixed effects (marginal): 0.65, variance explained by whole model (conditional): 0.81, ICC = 0.45. Model included data of: French provenances tested in Dijon, French and German provenances tested in Rostock.

|  | SSQ | MeanSQ | Numerator Degrees of Freedom | Denominator Degrees of Freedom | F | p |  | η² |
| --- | --- | --- | --- | --- | --- | --- | --- | --- |
| Species | 0.009 | 0.00058 | 15 | 162.0 | 18.40 | <0.001 | *** | 0.339 |
| Population | 0.000 | 0.00015 | 1 | 45.8 | 4.73 | 0.03 | * | 0.006 |
| Species : Population | 0.004 | 0.00023 | 15 | 175.6 | 7.38 | <0.001 | *** | 0.136 |
| Residuals | 0.013 |  |  |  |  |  |  |  |

Intra-specific variation was slightly smaller when seeds tested at another than their native location where included in the analysis, but inter-specific variation slightly higher than in the analysis in the main text.


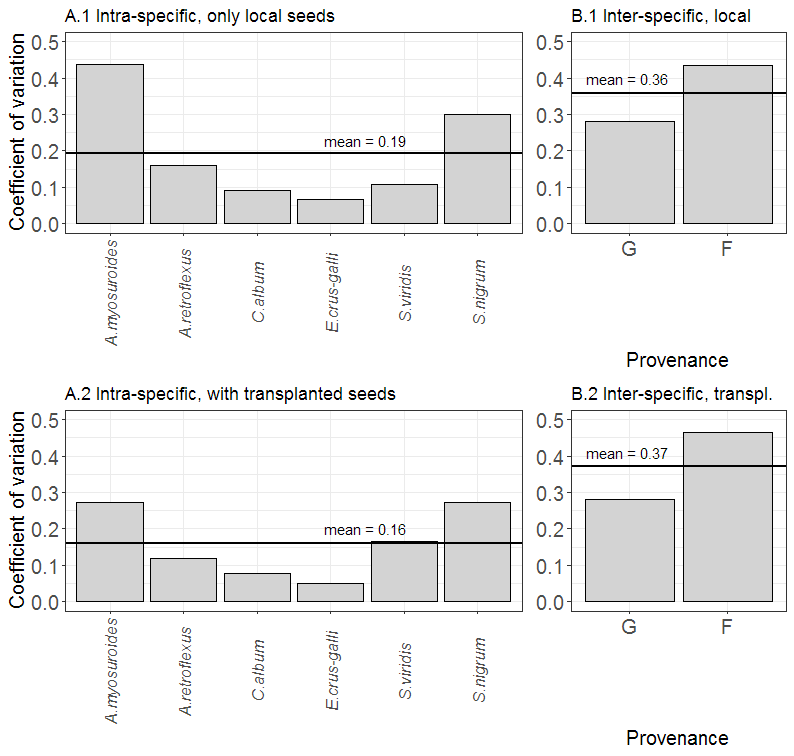


Figure 4: Components of variability in relative growth rate of six arable weed species from two provenances (G: German, F: French). A. Intra-specific variability (between populations). B. Inter-specific variability (within provenances). Comparison between two sets of experimental results: dataset 1 included only experiments with local seeds (Dijon –French provenances, Rostock – German provenances), dataset 2 additionally included experiments with transplanted seeds: French provenances tested in Rostock.

## Pairwise comparison of means between populations of the same species

Table 6: Pair-wise comparison of relative growth rate of 16 arable weed species between two populations (G: German, F: French). Includes only experiments with local seeds (Dijon –French provenances, Rostock – German provenances).

|  | Population | Relative growth rate [cm²*cm-2*°C-1*d-1] | Standard-error | diff G-F | df | T ratio | p |
| --- | --- | --- | --- | --- | --- | --- | --- |
| *Alopecurus myosuroides* | G | 0.0138 | 0.0038 | -0.00645 | 14.8 | -1.124 | 0.28 |
|  | F | 0.0202 | 0.0043 |  |  |  |  |
| *Amaranthus retroflexus* | G | 0.0274 | 0.0043 | 0.00481 | 11.5 | 0.943 | 0.37 |
|  | F | 0.0226 | 0.0027 |  |  |  |  |
| *Capsella bursa-pastoris* | G | 0.0207 | 0.0032 | 0.00369 | 13 | 0.671 | 0.51 |
|  | F | 0.0170 | 0.0045 |  |  |  |  |
| *Chenopodium album* | G | 0.0293 | 0.0046 | 0.00633 | 12.8 | 1.181 | 0.26 |
|  | F | 0.0230 | 0.0027 |  |  |  |  |
| *Digitaria sanguinalis* | G | 0.0294 | 0.0048 | -0.00947 | 17.6 | -1.598 | 0.13 |
|  | F | 0.0389 | 0.0035 |  |  |  |  |
| *Echinochloa crus-galli* | G | 0.0266 | 0.0049 | -0.00376 | 17.3 | -0.651 | 0.52 |
|  | F | 0.0304 | 0.0030 |  |  |  |  |
| *Galium aparine* | G | 0.0143 | 0.0038 | -0.00359 | 13.6 | -0.643 | 0.53 |
|  | F | 0.0179 | 0.0041 |  |  |  |  |
| *Geranium dissectum* | G | 0.0136 | 0.0039 | 0.00352 | 15.9 | 0.608 | 0.55 |
|  | F | 0.0100 | 0.0042 |  |  |  |  |
| *Matricaria chamomilla* | G | 0.0243 | 0.0034 | 0.00742 | 16.4 | 1.627 | 0.12 |
|  | F | 0.0169 | 0.0030 |  |  |  |  |
| *Matricaria inodora* | G | 0.0171 | 0.0037 | -0.00196 | 22.1 | -0.388 | 0.70 |
|  | F | 0.0190 | 0.0035 |  |  |  |  |
| *Poa annua* | G | 0.0108 | 0.0040 | -0.00607 | 21.6 | -1.21 | 0.24 |
|  | F | 0.0169 | 0.0030 |  |  |  |  |
| *Setaria viridis* | G | 0.0350 | 0.0046 | 0.01116 | 15.6 | 1.941 | 0.07 |
|  | F | 0.0239 | 0.0034 |  |  |  |  |
| *Solanum nigrum* | G | 0.0360 | 0.0046 | -0.01255 | 13 | -2.332 | **0.04** |
|  | F | 0.0485 | 0.0027 |  |  |  |  |
| *Sonchus asper* | G | 0.0161 | 0.0038 | -0.00389 | 20.3 | -0.796 | 0.44 |
|  | F | 0.0199 | 0.0031 |  |  |  |  |
| *Stellaria media* | G | 0.0185 | 0.0033 | 0.00427 | 10.1 | 0.827 | 0.43 |
|  | F | 0.0142 | 0.0040 |  |  |  |  |
| *Viola arvensis* | G | 0.0118 | 0.0033 | 0.00182 | 11.3 | 0.342 | 0.74 |
|  | F | 0.0100 | 0.0041 |  |  |  |  |

## Estimates of early relative growth rate per species

We pooled all available measurements for relative growth rate of individual plants from all experiments in Rostock and Dijon, fitted a linear mixed effects model, and estimated marginal means.

## Model used to estimate species means

Table 7: Species effects on early relative growth rate. Analysis of variance table of fixed effects for 16 species regardless of seed population. η²: proportion of variance accounted for by each of the main effects. ICC: Intraclass correlation coefficient - proportion of the variance explained by the grouping structure in the model (random effect). Variance explained by fixed effects (marginal): 0.55, variance explained by whole model (conditional): 0.74, ICC = 0.42. Model included data of: French provenances tested in Dijon, French and German provenances tested in Rostock, similar to Table 4 of the Supporting Information.

|  | SSQ | MeanSQ | Numerator Degrees of Freedom | Denominator Degrees of Freedom | F | p |  | η² |
| --- | --- | --- | --- | --- | --- | --- | --- | --- |
| Species | 0.019 | 0.00095 | 20 | 400.13 | 25.84 | <0.001 | *** | 0.549 |
| Residuals | 0.016 |  |  |  |  |  |  |  |

## Estimates

Table 8: Relative growth rates during seedling stage of 21 arable weed species, measured in two experiments in Dijon (France) and Rostock (Germany). Marginal means estimation from a mixed effects model, including experimental location and repetition as random factors.

| Species | Sample size (number of plants) | Experimental locations of data |  | Relative growth rate [cm²cm^-^²°C^-1^d^-1^] | SE |
| --- | --- | --- | --- | --- | --- |
| *Alopecurus myosuroides* | 18 | 2 |  | 0.0192 | 0.00223 |
| *Amaranthus retroflexus* | 35 | 2 |  | 0.0238 | 0.00191 |
| *Apera spica-venti* | 3 | 1 (Rostock) |  | 0.0229 | 0.00268 |
| *Capsella bursa-pastoris* | 24 | 2 |  | 0.0132 | 0.00396 |
| *Centaurea arvensis* | 6 |  |  | 0.0206 | 0.00204 |
| *Chenopodium album* | 38 | 2 |  | 0.0141 | 0.00304 |
| *Digitaria sanguinalis* | 25 | 2 |  | 0.0244 | 0.00189 |
| *Echinochloa crus-galli* | 17 | 2 |  | 0.0367 | 0.00216 |
| *Galium aparine* | 24 | 2 |  | 0.0288 | 0.00224 |
| *Geranium dissectum* | 17 | 2 |  | 0.0177 | 0.00224 |
| *Anchusa arvensis* | 9 | 1 (Rostock) |  | 0.0118 | 0.00228 |
| *Matricaria chamomilla* | 22 | 2 |  | 0.0196 | 0.00197 |
| *Matricaria inodora* | 26 | 2 |  | 0.0184 | 0.00208 |
| *Papaver rhoeas* | 12 | 1 (Rostock) |  | 0.0186 | 0.00264 |
| *Poa annua* | 28 | 2 |  | 0.0161 | 0.00199 |
| *Setaria viridis* | 33 | 2 |  | 0.0270 | 0.00207 |
| *Solanum nigrum* | 36 | 2 |  | 0.0446 | 0.00196 |
| German population |  | 1(Rostock) |  | 0.0360 | 0.00460 |
| French population |  | 1(Dijon) |  | 0.0485 | 0.00270 |
| *Sonchus asper* | 20 | 2 |  | 0.0201 | 0.00208 |
| *Stellaria media* | 45 | 2 |  | 0.0157 | 0.00192 |
| *Veronica hederifolia* | 10 | 1 (Rostock) |  | 0.0101 | 0.00389 |
| *Viola arvensis* | 25 | 1 (Rostock) |  | 0.0116 | 0.00202 |

References

1. Colbach N, Moreau D, Dugué F, Gardarin A, Strbik F, Munier-Jolain N. The response of weed and crop species to shading. How to predict their morphology and plasticity from species traits and ecological indexes. European Journal of Agronomy. in rev.
