# Supplementary material for: Populations of arable weed species show intra-specific variability in germination base temperature but not in early growth rate
Source: PLoS One. 2020 Oct 9;15(10):e0240538. doi: 10.1371/journal.pone.0240538 (PMC7546504; doi:10.1371/journal.pone.0240538)
Supplement: Attachment — Submitted filename: PONE-D-20-16133_reviewer2_Response.pdf [file pone.0240538.s002.pdf]

PLOS ONE

Arable weed species show local adaptation in germination base temperature but not in seedling growth rate

--Manuscript Draft--

**Manuscript Number:**	PONE-D-20-16133
**Article Type:**	Research Article
**Full Title:**	Arable weed species show local adaptation in germination base temperature but not in seedling growth rate
**Short Title:**	Arable weed adaptation
**Corresponding Author:**	Jana Bürger<br>Universitat Rostock Agrar- und Umweltwissenschaftliche Fakultat<br>Rostock, GERMANY
**Keywords:**	weed biology; early growth; agriculture; agroecology; modelling; germination trait; plant trait
**Abstract:**	Trait-based research can improve our understanding and projections of weed population dynamics, aiding environmentally friendly weed management. Depending on scale and scope of a study, intra-specific trait variability may be larger than previously assumed, and should then be considered for inclusion into the research project. We analysed the intra-specific variability of two key traits in the population dynamics of arable weed species. Germination base temperature and the seedlings' early relative growth rate were determined in climate chamber and green house experiments. Then we analysed how climate of seed origin influenced the results. Germination base temperatures were higher in seeds of the same species stemming from colder climate. Seedling relative growth rates did not differ between seed provenances. Species with a higher germination base temperature had a higher relative growth rate. Models simulating weed growth should therefore reflect the differences in germination traits among populations, especially when they are used for studies of weed community assemblies in a local to regional extent.
**Order of Authors:**	Jana Bürger
	Nathalie Colbach
	Andrey V. Maleyshev
**Additional Information:**	
**Question**	**Response**
**Financial Disclosure**<br><br>Enter a financial disclosure statement that describes the sources of funding for the work included in this submission. Review the submission guidelines for detailed requirements. View published research articles from *PLOS ONE* for specific examples.<br><br>This statement is required for submission and **will appear in the published article** if the submission is accepted. Please make sure it is accurate.	JB was funded by Deutsche Forschungsgemeinschaft (https://www.dfg.de/) under the grant number BU 3097/1-1. NC acknowledges past and current funding by INRAE. The funders had no role in study design, data collection and analysis, decision to publish, or preparation of the manuscript.

Powered by Editorial Manager® and ProduXion Manager® from Aries Systems Corporation

**Unfunded studies**<br>Enter: *The author(s) received no specific funding for this work.*<br><br>**Funded studies**<br>Enter a statement with the following details:<br>• Initials of the authors who received each award<br>• Grant numbers awarded to each author<br>• The full name of each funder<br>• URL of each funder website<br>• Did the sponsors or funders play any role in the study design, data collection and analysis, decision to publish, or preparation of the manuscript?<br>• **NO** - Include this sentence at the end of your statement: *The funders had no role in study design, data collection and analysis, decision to publish, or preparation of the manuscript.*<br>• **YES** - Specify the role(s) played.<br><br>* typeset	
**Competing Interests**<br><br>Use the instructions below to enter a competing interest statement for this submission. On behalf of all authors, disclose any competing interests that could be perceived to bias this work—acknowledging all financial support and any other relevant financial or non-financial competing interests.<br><br>This statement **will appear in the published article** if the submission is accepted. Please make sure it is accurate. View published research articles from *PLOS ONE* for specific examples.	The authors have declared that no competing interests exist.

**NO authors have competing interests**<br><br>Enter: *The authors have declared that no competing interests exist.*<br><br>**Authors with competing interests**<br><br>Enter competing interest details beginning with this statement:<br><br>*I have read the journal's policy and the authors of this manuscript have the following competing interests: [insert competing interests here]*<br><br>* typeset	
**Ethics Statement**<br><br>Enter an ethics statement for this submission. This statement is required if the study involved:<br><br>• Human participants<br>• Human specimens or tissue<br>• Vertebrate animals or cephalopods<br>• Vertebrate embryos or tissues<br>• Field research<br><br>Write "N/A" if the submission does not require an ethics statement.<br><br>General guidance is provided below. Consult the submission guidelines for detailed instructions. **Make sure that all information entered here is included in the Methods section of the manuscript.**	N/A

**Format for specific study types**<br><br>**Human Subject Research (involving human participants and/or tissue)**<br>• Give the name of the institutional review board or ethics committee that approved the study<br>• Include the approval number and/or a statement indicating approval of this research<br>• Indicate the form of consent obtained (written/oral) or the reason that consent was not obtained (e.g. the data were analyzed anonymously)<br><br>**Animal Research (involving vertebrate animals, embryos or tissues)**<br>• Provide the name of the Institutional Animal Care and Use Committee (IACUC) or other relevant ethics board that reviewed the study protocol, and indicate whether they approved this research or granted a formal waiver of ethical approval<br>• Include an approval number if one was obtained<br>• If the study involved *non-human primates*, add *additional details* about animal welfare and steps taken to ameliorate suffering<br>• If anesthesia, euthanasia, or any kind of animal sacrifice is part of the study, include briefly which substances and/or methods were applied<br><br>**Field Research**<br><br>Include the following details if this study involves the collection of plant, animal, or other materials from a natural setting:<br>• Field permit number<br>• Name of the institution or relevant body that granted permission	
**Data Availability**<br><br>Authors are required to make all data underlying the findings described fully available, without restriction, and from the time of publication. PLOS allows rare exceptions to address legal and ethical concerns. See the PLOS Data Policy and FAQ for detailed information.	Yes - all data are fully available without restriction

A Data Availability Statement describing where the data can be found is required at submission. Your answers to this question constitute the Data Availability Statement and **will be published in the article**, if accepted.<br><br>**Important:** Stating 'data available on request from the author' is not sufficient. If your data are only available upon request, select 'No' for the first question and explain your exceptional situation in the text box.<br><br>Do the authors confirm that all data underlying the findings described in their manuscript are fully available without restriction?	
**Describe where the data may be found in full sentences. If you are copying our sample text, replace any instances of XXX with the appropriate details.**<br><br>• If the data are **held or will be held in a public repository**, include URLs, accession numbers or DOIs. If this information will only be available after acceptance, indicate this by ticking the box below. For example: *All XXX files are available from the XXX database (accession number(s) XXX, XXX.).*<br>• If the data are all contained **within the manuscript and/or Supporting Information files**, enter the following: *All relevant data are within the manuscript and its Supporting Information files.*<br>• If neither of these applies but you are able to provide **details of access elsewhere**, with or without limitations, please do so. For example:<br><br>*Data cannot be shared publicly because of [XXX]. Data are available from the XXX Institutional Data Access / Ethics Committee (contact via XXX) for researchers who meet the criteria for access to confidential data.*<br><br>*The data underlying the results presented in the study are available from (include the name of the third party*	All data has been uploaded to the DRYAD repository. doi:10.5061/dryad.nzs7h44p0

and contact information or URL).<br>• This text is appropriate if the data are owned by a third party and authors do not have permission to share the data.<br><br>* typeset	
Additional data availability information:	Tick here if the URLs/accession numbers/DOIs will be available only after acceptance of the manuscript for publication so that we can ensure their inclusion before publication.

Powered by Editorial Manager® and ProduXion Manager® from Aries Systems Corporation

Manuscript
Click here to access/download;Manuscript;Manuscript-re1.docx

Arable weed species show local adaptation in germination base temperature but not in seedling growth rate

Jana Bürger [1*], Andrey V. Malyshev [2], Nathalie Colbach[3]

1 Crop Health, Faculty of Agricultural and Environmental Sciences, University of Rostock, Rostock, Germany

2 Experimental Plant Ecology, Institute of Botany and Landscape Ecology, University of Greifswald, Greifswald, Germany

3 Agroécologie, AgroSup Dijon, INRAE, Univ. Borgogne, Univ. Bourgogne Franche-Comté, Dijon, France

*Corresponding author

E-mail: jana.buerger@uni-rostock.de (JB)

Abstract

Trait-based research can improve our understanding and projections of weed population dynamics, aiding environmentally friendly weed management. Depending on scale and scope of a study, intra-specific trait variability may be larger than previously assumed, and should then be considered for inclusion into the research project. We analysed the intra-specific variability of two key traits in the population dynamics of arable weed species. Germination base temperature and the seedlings' early relative growth rate were determined in climate chamber and green house experiments. Then we analysed how climate of seed origin influenced the results. Germination base temperatures were higher in seeds of the same species stemming from colder climate. Seedling relative growth rates did not differ between seed provenances. Species with a higher germination base temperature had a higher relative growth rate. Models simulating weed growth should therefore reflect the differences in germination traits among populations, especially when they are used for studies of weed community assemblies in a local to regional extent.

Keywords: weed biology, early growth, agriculture, agroecology, modelling

Introduction

Over the last decades, there has been an increase in trait-based analyses and modelling, both in ecology in general [1] and in weed ecology in particular [2,3] followed by a debate on the importance and implications of intraspecific trait variability. Initially, the assumption of many trait-based approaches was that intraspecific variability is negligibly small compared to the interspecific (between-species) variability, and that species can be characterised by mean trait values [4]. This has since been challenged by a number of studies [5,6]. Trait-based research projects therefore need to critically consider whether to include intra-specific trait variability (ITV) in their set-up, based on species, scale, and scope of the study [7].

The mechanisms that cause intra-specific variability as a species' response to its environmental conditions include adaptation (genetic variability) and phenotypic plasticity [8]. They are expressed in a range of functional plant and seed traits [4,9]. Functional traits have been defined as measurable features which interact with ecological factors through specific functions in order to explain plant fitness components like growth, reproduction and survival [9,10]. Sometimes, they are classified as hard and soft traits. A hard, usually physiological trait would be a feature which is difficult to quantify, but describes a plant function more accurately in contrast to more easily measured, often morpho-anatomical soft traits that can sometimes provide proxies for a hard trait [11,12].

Intraspecific variability arises at different scales and ecological organisational levels. It can be decomposed into parts [7]: the *between-individual variability* is the trait variability within a given population. The difference in trait values between populations can be called the *population-level variability* or *between-population variability*. A population in this context could be any group of sample individuals connected by a common environment: groups of plants growing in distinct places (geographical regions, different habitats or even parts of a field) or otherwise groups of plants growing at the same location but from seed samples sourced in different places (e.g. a greenhouse or field experiment comparing different seed provenances).

The study of traits in weed research aims to understand which processes drive weed community composition and population dynamics. One of the main goals is to contribute to a more environmentally-friendly weed management [2,13]. This includes attempts to make the modelling of weed populations generic rather than species-specific, based on a mechanistic representation of processes and involved species traits [3,14,15].

Arable fields, compared to other habitats of wild plant species, are characterised by a high frequency of disturbances through and strong competition from crops. The right timing of germination and establishment together with the ruderal growth and regeneration strategy are therefore of special importance for plant establishment and fitness in weed plants [16,17].

Selection pressure favours species' responses to local environmental cues that synchronise germination with periods that are optimal for seedling survival and establishment as well as reproduction [18–20]. Likewise, plants which compete better for light, nutrients and water have a higher fitness (i.e. the number of seeds produced per germinated seed), are more tolerant to resource deficits, or have higher plasticity in response to stresses [21].

The high level of disturbance and the ruderal strategy, suggest a high ability of adaptation in weed species, and consequently a high intra-specific trait variability. This must be represented in trait-based approaches in weed ecology to make them widely usable and valid in a more general context. Existing trait databases are extensive sources for trait information, but they are usually of limited use when it comes to intraspecific trait variation under variable environmental settings [1,3].

Only a small number of studies investigated intra-specific trait variability in arable weeds, mainly for soft traits like canopy height, specific leaf area, or biomass. These studies concentrated on trait variability as a response to different cropping systems, or explore the effect of variability on community assembly patterns and on ecosystem functioning [22–24].

Even less studies analysed intra-specific variability in traits associated more to processes in population dynamics, like germination and early growth. In this paper, we aim to close this gap and analyse the intra-specific variability of two key traits in arable weed species: germination base temperature and early relative growth rates. Base temperature has been identified as one key parameter necessary for modelling weed seed germination [25] determining the days where germination is possible as well as controlling the speed of germination, emergence and post-emergence growth. The latter also depends on the early growth rate of seedlings which is an important ecophysiological trait in crop/weed competition models [26].

Results of earlier studies were not consistent on the extent of intra-specific variability in these two traits, or other traits associated to germination [27–31]. Differences between populations were found for some species, not for others not. It is possible that the population effect is only evident

when populations come from locations with sufficiently strong differences in environmental conditions. No differences were found between populations from near-by regions like Northern vs. Eastern France [32] and Central vs. Northern Italy [33,34].

In our study, we therefore examine experimental results obtained with seeds from two regions with contrasting environmental conditions. We hypothesised that between-population differences in germination base temperature and seedling relative growth rate exist. We expected that base temperatures as well as early relative growth rates would be lower in the location with colder climate and lower solar radiation, due to lower resource availability.

Materials and Methods

Two experiments were carried out in Rostock (Germany) between 2016 and 2017 following the protocols of earlier experiments carried out in Dijon (France) between 2009 and 2012 [14,21,35].

Experiment on germination base temperature

Seed germination of 13 species was tested at four to six constant temperatures. Seeds were laid out in petri dishes (Ø 9 cm) lined with a double layer of filter paper and moistened with 5 ml deionised water, in some cases with 5 ml solution of KNO_3 (10 mmol) to break seed dormancy. Four replicates of 50 or 100 seeds per treatment were placed in temperature chambers with a 12-hour photoperiod. Petri dishes were checked for germination at least once a day and moistened when the filter paper started to fall dry. Seeds were considered germinated once a radicle was clearly visible. Experiments lasted for approx. 4 weeks each, or until no more germination occurred during 7 days. Temperature in the chambers was monitored with data loggers (HOBO UX100-001/ Voltcraft PL-125-T2) every 10 minutes. The average of all measurements during the experiment was used for calculations.

Time-to-event models were fitted to the germination data [36]. We used a nonlinear and asymmetric three-parameter Weibull function following the equation

$$GP_t = GP_{final} \times \left(1 - e^{-e^{b\times(\ln(t) - \ln(i))}}\right) \; for\; t > 0$$

$$GP_t = 0 \; for\; t = 0 \quad (1)$$

where t is the time (in days) since water addition, GP_t the proportion of seeds germinated up to the sampling time t, GP_{final} the final proportion of seeds germinated out of the sample, i the point when inflection of the curve happens and b the shape parameter reflecting the steepness of the curve. We calculated t_{50}, which is the time to reach 50% of the final germination proportion from the fitted model parameters.

Germination rates (GR) were calculated as the inverse of t_{50}: $GR = \frac{1}{t_{50}}$ (2). A linear regression of the germination rate (in d^{-1}) over temperature (T in °C) was fitted per species: $GR = a + b \times T$ (3). The base temperature (T_b, in °C) was determined as the x-intercept of this regression line and calculated as $T_b = -\frac{a}{b}$ (4) with a and b being the intercept and slope of the regression [37].

Experiment on relative growth rate in seedling stage

Early growth was monitored for 21 weed species in several experimental trials between spring 2016 and spring 2017 (Table 1), using the method developed by Colbach et al. [21]. Each trial included species that would germinate approximately around the same time in the field, i.e. spring germinating species in March/ April, early summer germinating species in May/ June and autumn germinating species in September/October. *V. arvensis* and *G. aparine* were tested both in spring and in autumn. The experiment included seeds from populations in Northern Germany for all 21 species. For five summer-germinating species and one autumn-germinating species, seeds from French populations were tested, additionally.

Seeds were placed in Petri dishes, wetted with water or a KNO_3 solution and were set to germinate under optimal temperature and light conditions. When germination started, 20 seeds per species were placed in individual pots with standard garden earth, in a depth of 2 - 20 mm, depending on seed size, i.e. large seeds were sown deeper. Pots were placed in an unheated green house, with

additional light (Philips sodium vapour lamps, 400 W) to maintain a 12-hour photoperiod. For six species (*A. retroflexus*, *Matricaria chamomilla*, *S. nigrum*, *S.media*, *V. hederifolia, V. arvensis*) the number of plants which initially survived in the pots was not sufficient for further analyses, therefore another 20 plants these species were grown within two weeks after the start of the experimental run. Temperature was recorded with data loggers (HOBO UX100-001/ Voltcraft PL-125-T2) every 15 minutes and averaged over the day. Pots were watered regularly, keeping the soil constantly moist to ensure non-limited growth.

Leaf area development was monitored by a non-destructive approach. One photograph was taken of each plant per day, with a red cardboard reference of pre-defined area (1cm² or 4cm²) placed beside the pot at soil height. Leaf area was estimated with Easy Leaf Area software [38] from the green pixels on the picture. Some of the 20 plants were harvested at 2-leaf, 4-leaf and 6-leaf stage respectively and their true leaf area determined using a flat bed desktop scanner (CanoScan LiDE 220). The ratio of leaf area on photograph to leaf area on scan was calculated per plant. A linear regression was fitted per species to this ratio over thermal time after emergence. Finally, the leaf area measurements from photographs of the remaining plants were corrected using the estimated regression function.

The following equation was fitted to model leaf area development of each plant grown to the end of the experiment

$$LA = LA_0 \times e^{RGR \times TT_d} \qquad (5)$$

where LA is leaf area (cm²) of the plant on sampling date d, LA_0 is (initial) leaf area at emergence (cm²) , TT_d is thermal time from emergence to sampling day d (°C·d), and RGR the relative growth rate ($cm^2 \cdot cm^{-2} \cdot °C^{-1} \cdot d^{-1}$). Leaf area was $\log_n$-transformed and parameters fitted by linear regression over TT_d. Data points beyond the phase of exponential growth were left out of analysis. The end of this period was determined as the date when the local slope of $\log_n$(LA) vs TT_d dropped below 1/10 of

the slope at emergence (when TT_d=0). Local slopes were calculated as the derivate of a polynomial fitted to log_n(LA) vs TT_d (S1 Fig.).

Thermal time TT_d was calculated as

$$TT_d = \sum_{i=0}^{d}(T_i - T_b)\ for\ T_i \geq T_b \qquad (6)$$

where T_i is the mean temperature of day i (°C) and T_b is the species-specific base temperature (°C). For twelve species, we used the germination base temperature obtained in our own experiment to calculate TT_d. For the other nine species we used literature values. Only plants for which the R^2 of the regression (equ. 5) exceeded 0.66 were used for further analyses to eliminate bad fits. For the calculation of averages per-plant-values were weighted by the inverse of the relative standard error of each plant (i.e. $se\_LA0_p/LA0_p$ and se_RGR_p/RGR_p, with se_LA0_p and se_RGR_p the standard errors estimated from equ. 5).

Species choice and seed material

For the experiments in Rostock we focused on the most frequent and important weeds in Northern German arable fields (Table 1). Seeds were mostly sourced from the experimental gardens and experimental fields of Rostock University, but in a few cases obtained from commercial suppliers aiming for seed lots from Northern Germany (S1 Table). The seeds from French provenance used in the experiment on relative growth rate were provided by the INRA weed seed collection (UMR Agroécologie, INRA Dijon). Seeds were stored in paper bags at room temperature and subjected to cold stratification prior to the experiments if necessary to break dormancy.

Rostock/ Germany (54° 5′ N, 12° 8′ E, 14m asl) is located approximately 1200km northeast of Dijon/ France (47° 19′ N, 5° 3′ E, 257m asl). The climate in Rostock is characteristic for the transition zone between oceanic and continental climates with a mean temperature of 8.4°C and 591 mm precipitation. It has long winters with short periods of strong frost, a warm summer and a precipitation pattern with low amounts in spring and autumn and a maximum in July.

Dijon, in contrast has an oceanic climate with a continental tendency. Annual mean temperature is 10.5°C, with cold winters and hot summers. The precipitation of 732 mm is distributed nearly uniformly over all months, with a small maximum in May. Rostock has a shorter growing period, more days with a water deficit (evapotranspiration > precipitation) in spring and a higher probability of late frosts [39].

Mean air temperature is lower in Rostock than Dijon between January and October and similar at the end of the year (Figure 1). In contrast, soil temperatures are similar between mid May and August, but considerably lower in Rostock outside of summer.

Figure 1: Average daily temperatures in France (Epoisses near Dijon) and Germany (Warnemünde near Rostock) between 2000 and 2019. A. Mean air temperature (2m above ground). B. Minimum air temperature (2m above ground). C. Mean soil temperature (10cm below ground). GAM smoothing over daily values, grey area is 95% confidence interval. Weather stations at the INRA experimental station of Dijon-Epoisses, and German Weather Service (DWD) Rostock-Warnemünde (Datasources: platform INRA CLIMATIK, www.opendata.dwd.de).

Table 1: Species included in experiments on germination base temperature and early growth rate. The paper reports on experimental results from Rostock, and uses additional results from earlier experiments in Dijon to analyse intra-specific variability. Meaning of symbols: "x": species was tested, "-": species was not tested.

Experiment	Base temperature		Relative growth rate	Relative growth rate	Relative growth rate
Experimental location	Rostock		Rostock	Rostock	Dijon
Seed provenance	German		German	French	French
Species		Season of experiment in Rostock			
Alopecurus myosuroides	x	Autumn	x	x	x
Amaranthus retroflexus	-	Early summer	x	x	x
Anchusa arvensis	x	Spring	x	-	-

Apera spica-venti	x	Autumn	x	-	-
Capsella bursa-pastoris	-	Spring & Autumn	x	-	x
Centaurea arvensis	x	Spring	x	-	x
Chenopodium album	x	Early summer	x	x	x
Digitaria sanguinalis	-	Early summer	x	-	x
Echinochloa crus-galli	x	Early summer	x	x	x
Galium aparine	-	Autumn	x	-	x
Geranium dissectum	x	Spring	x	-	x
Matricaria chamomilla	x	Spring	x	-	x
Matricaria inodora	x	Spring	x	-	x
Papaver rhoeas	x	Spring	x	-	-
Poa annua	-	Autumn	x	-	x
Setaria viridis	x	Early summer	x	x	x
Solanum nigrum	-	Early summer	x	x	x
Sonchus asper	-	Autumn	x	-	x
Stellaria media	x	Spring	x	-	x
Sisymbrium officinale	x	Autumn	-	-	-
Veronica hederifolia	-	Spring	x	-	-
Viola arvensis	-	Spring & Autumn	x	-	x

Analysis of inter-specific and intra-specific variability

Data

To analyse inter-specific and intra-specific variation of the two plant traits, we pooled the results of the described experiments in Rostock with results of similar experiments obtained in Dijon (Table 2). The Dijon experiments were carried out on weed species frequently found in arable crops in temperate France, choosing species that differed in terms of clade, emergence season, seed mass [14,21]. Here, we only use data from Dijon for species that are also important in Northern Germany.

For clarity, “Rostock” and “Dijon” will refer to the locations of experiments while “French” and “German” will refer to seed populations originating from the two different regions.

Table 2: Overview of data used for analysis of inter-specific and intra-specific variability. All data from Dijon was selected to match the species tested in Rostock.

	Seed provenance	Experimental location	
		Rostock	Dijon
Base temperature experiment	German	13 species	-
	French	-	6 species
Relative growth rate experiment	German	21 species	-
	French	6 species	16 species

We included four sources of variation into our analysis: 1. species (=inter-specific variability), 2. seed provenance (= between-population variability), 3. differences in the experimental conditions between experimental locations (contributing to between-individual variability), and for the growth rate experiment 4. differences in the experimental conditions between repetitions within a location (also contributing to between-individual variability).

Inter-specific and intra-specific variability in germination base temperature

The effects of species and population on germination base temperature were tested with a two-way Anova. Pairwise tests were run on germination base temperatures between populations of the same species, comparing the estimates by calculating the z-score

$$z = \frac{T_{bG} - T_{bF}}{\sqrt{se\_T_{bG}^2 + se\_T_{bF}^2}} \qquad (7)$$

where T_{bG} and T_{bF} are the germination base temperature estimates and se_T_{bG} or se_T_{bF} the corresponding standard errors for German and French populations, respectively. The significance of the difference was calculated as

$$p = 1 - 2\int_{-\infty}^{z} \frac{1}{\sqrt{2\pi}} e^{\frac{-u^2}{2}} du \qquad (8).$$

Inter-specific and intra-specific variability in relative growth rate

Mixed effect models were used to analyse early relative growth rates with species, seed population and experimental location as fixed factors. We included repetition as a random factor to account for the nested structure of the data and the unbalanced number of plants (3 to 10) within each combination of species, population, location, and repetition [40]. A Type III Anova with Satterthwaite's method for denominator degrees of freedom and F-statistic was used to test the significance of effects of species, population and experimental location.

We also compared intra-specific and inter-specific variability by means of variation coefficients. First, we averaged measured relative growth rates per species and population. Then we calculated the coefficient of variation between population means per species (intra-specific variation) or between species means per population (inter-specific variation).

We estimated marginal means to specify the relative growth rate per species and population and conducted a pair-wise comparison of means to test for differences between populations. If the difference was not significant, we estimated the species relative growth rate from both populations combined.

Within the paper, we present analyses of relative growth rate variability without the data collected from French seeds in the Rostock experiment. The results differed only slightly when incorporating these "transplanted seeds" and are included in the Supporting information. We decided against using this data in the main analysis because it introduced additional variability and further un-balance to our analysis (by growing seeds in an environment different to their origin and only transplanting one population, not both).

Relationship between base temperature and relative growth rate

We tested the relation between base temperature, provenance and early relative growth rate (RGR) by fitting a linear model including all species with RGR for both provenances, followed by an ANOVA.

Software

All statistical analyses were carried out with R 3.1.6 [41], germination curves fitted with package *drc* [42], linear mixed models fitted with packages *lme4* [43] and *lmerTest* [44], and marginal means estimated using package *emmeans* [45].

Results

Germination base temperatures

All regressions correlating germination rates to temperatures had good fits, with R^2 ranging from 0.81 to 0.99 (Table 3). Base temperatures varied between -0.2°C (*Apera spica-venti*) and 11.9°C (*Setaria viridis*).

Table 3: Germination base temperature of 13 weed species from German seed populations. Values are estimates +/- standard error. Comparison to values from French provenance and/or literature. Significant differences in bold type. Main emergence periods given for Northern Germany (G) and Eastern France (F). Periods are similar for both provenances if not marked otherwise.

Species	Main emergence periods in fields	Base temperature (°C) – German provenance	R^2	Base temperature (°C) – French provenance	p-value of difference	Ranges of base temperature from other sources (°C)
Alopecurus myosuroides	G: autumn<br>F: autumn & spring	1.9 ± 0.7	0.95	0.0 [32]	0.31	-
Amaranthus retroflexus	early summer	10.5 ± 1.3	0.86	8.9 ± 1.1 [35]	0.35	10-12.6 [46] [34], [47]
Anchusa arvensis	G: spring & autumn	8.3 ± 2.0	0.81	-		
Apera spica-venti	G: Early spring & autumn	-0.2 ± 0.9	0.92	-		
Centaurea arvensis	Autumn	3.3 ± 1.0	0.81	2.2 ± 1.2 [48]	0.48	1.1-1.9 [29]
Echinochloa crus-galli	early summer	10.0 ± 0.6	0.92	6.2 ± 0.6 [35]	**< 0.001**	10.4-13.2 [47], [34] [49]
Geranium dissectum	G: spring & autumn<br>F: autumn	4.0 ± 0.4	0.93	0 ± 1.3 [35]	**0.003**	-

Matricaria chamomilla	G: spring & autumn	4.7 ± 0.6	0.96	-		-
Matricaria inodora	spring & autumn	2.9 ± 0.5	0.96	2.0 ± 0.6 [35]	0.25	-
Papaver rhoeas	G: spring & autumn	2.5 ± 0.2	0.99			1.0 Spain [50]
Setaria viridis	early summer	11.9 ± 0.5	0.96	-		6.1-12.5 [47], [51]
Sisymbrium officinale	G: autumn	5.7 ± 0.2	0.96	-		
Stellaria media	G: all year<br>F: spring & autumn	2.3 ± 0.2	0.98			1.4 [52]

For the six species for which we had measurements for both seed populations, the base temperatures for the German populations were all clearly higher than for the French populations (Figure 2), with a significant difference for two species (Table 3). In an analysis of variance for the same data, species and population both had significant effects on germination base temperature (partial R^2=0.88, p = 0.001 and partial R^2 = 0.10, p = 0.012 respectively). The variability in base temperature was higher among species than between populations.

Figure 2: Germination base temperatures of six weed species comparing two populations from contrasting provenances. The dashed line marks the ratio 1:1. French populations tested in Dijon, German populations in Rostock, using growth chambers with similar conditions in both cases.

Relative growth rates

Relative growth rates were strongly dependent on species which accounted for about a third of the variation (η^2 = 0.303, p<0.001). Provenance of seeds had no effect in itself (p= 0.93), but we found a significant species:provenance interaction (η^2 = 0.151, p <0.001) accounting for half as much variation as species. Differences in the experimental conditions (environment, equipment and materials, handling) between the several runs explained nearly half the variance in the data (Intra-

correlation Coefficient ICC = 0.47). This variation could be attributed mainly to the repetitions. Experimental location had no significant effect (Table 2 in Supp. Inf. S1 File).

Variation in relative growth rate between species was about three times the mean variation within species (Figure 3). Species-specific relative growth rates varied between 0.0101 (*V. hederifolia*) and 0.0446 $cm^2cm^{-2}°C^{-1}d^{-1}$ (*S. nigrum*) (Table 5). The pairwise comparison of means between populations for each species showed a significant difference of relative growth rate only for one species, *S. nigrum* (Table 4 in Supp Inf. S1 File).

Figure 3: Components of variability in relative growth rate of 16 arable weed species. A. Intra-specific variability: coefficient of variation among population averages of all plants from the same species. B. Inter-specific variability: coefficient of variation between species averages of all plants from the same provenance. One population per species and provenance : G: German, F: French.

Table 4: Relative growth rates during seedling stage of 21 arable weed species, measured in two experiments in Dijon (France) and Rostock (Germany). Marginal means estimation from a mixed effects model, including experimental location and repetition as random factors.

Species	Sample size (number of plants)	Experimental locations of data	Relative growth rate [$cm^2cm^{-2}°C^{-1}d^{-1}$]	SE
Alopecurus myosuroides	18	2	0.0192	0.00223
Amaranthus retroflexus	35	2	0.0238	0.00191
Apera spica-venti	3	1 (Rostock)	0.0229	0.00268
Capsella bursa-pastoris	24	2	0.0132	0.00396
Centaurea arvensis	6		0.0206	0.00204
Chenopodium album	38	2	0.0141	0.00304
Digitaria sanguinalis	25	2	0.0244	0.00189
Echinochloa crus-galli	17	2	0.0367	0.00216
Galium aparine	24	2	0.0288	0.00224
Geranium dissectum	17	2	0.0177	0.00224
Anchusa arvensis	9	1 (Rostock)	0.0118	0.00228
Matricaria chamomilla	22	2	0.0196	0.00197
Matricaria inodora	26	2	0.0184	0.00208
Papaver rhoeas	12	1 (Rostock)	0.0186	0.00264

Poa annua	28	2	0.0161	0.00199
Setaria viridis	33	2	0.0270	0.00207
Solanum nigrum	36	2	0.0446	0.00196
German population		1(Rostock)	0.0360	0.00460
French population		1(Dijon)	0.0485	0.00270
Sonchus asper	20	2	0.0201	0.00208
Stellaria media	45	2	0.0157	0.00192
Veronica hederifolia	10	1 (Rostock)	0.0101	0.00389
Viola arvensis	25	1 (Rostock)	0.0116	0.00202

Relationship between base temperature and growth rate

We found a positive relationship between germination base temperature and early relative growth rate in the studied species. A linear model for the French populations had a fit of $R^2 = 0.74$ and for German populations of $R^2 = 0.85$. The slopes were not significantly different when testing for the influence of seed origin on the relation between the two variables ($t = -2.022$, $p > 0.05$). Species, which germinate at higher temperatures, grow more per degree-day than species with low germination base temperatures.

Figure 4: Relationship between germination base temperature and relative growth rate per species and population. Fitted functions: German seeds: RGR = 0.011 + 0.0019 x T_b, R^2=0.86; French seeds: RGR = 0.012 + 0.0023 x T_b, R^2 = 0.74.

Discussion

We reported on the outcome of two germination and early growth experiments. We compared the results to the outcome of similar experiments with seeds from contrasting climate conditions. The climatic gradient between the seed provenances also causes differences between the regionally adapted agricultural management systems.

Germination base temperatures are higher in colder climate populations

Our results on base temperature of weed species from Northern German provenance are in good correspondence with the ranges reported for European arable weed species in the literature before (Table 3). Generally, we found that for a given species, base temperatures were higher for seeds originating from the colder climate. At first sight, this result is slightly surprising: we expected base temperatures to decrease as an adaptation to lower soil temperatures in Rostock compared to Dijon. A negative relation between ambient temperature and germination temperature requirements of populations of the same species, though, has been found by other authors as well, both for a North-South gradient [53] and an altitudinal gradient [54]. The germination base temperature seems to be adapted generally in a way to prevent seeds from germinating in unfavourably cold conditions. The intuitively expected relationship of higher environmental temperatures leading to higher germination temperature requirements was observed for many organismal groups [55]. For our experiments, it is found at the inter-specific level: typical summer-germinating weed species originate from warmer regions and their base temperature is a considerably higher than for the spring and autumn germinating species.

Possibly, our results were also influenced by the environmental conditions at seed set and during seed development of our samples. Such effects can influence dormancy release, seed quality or germination percentages at different temperatures in germination experiments [53,56]. Differences in seed germination behaviour have been reported for spring vs autumn germinated plants [32,57], for varying seed positions on the same plant, for species with seed polymorphism like *C. album* [58], and for populations with differing life cycle strategies growing side by side [59].

However, it would be necessary to use a common garden approach in order to distinguish between plastic responses of different provenances vs. genetic adaptation [30,33,60]. As we compared only two populations of each species, it is not possible to calculate an exact difference of base temperatures between French and German populations, as a generally applicable value.

Base temperature may control germination only for spring emergence periods

Germination base temperature has been generally assumed as giving a cue of favourable growing conditions for seeds to start germination and emergence, but also halt germination in autumn before the seedlings' ability to survive winter could be decreased by late germination [57,60,61]. Our experiments suggest that this relationship may only hold true for the spring emergence periods and therefore for species that are facultative or strictly spring-germinating. In a previous analysis, an influence of base temperature could only be found on the onset of the spring emergence period but not the onset of autumn emergence or end of either of these [14].

Figure 5 gives the main emergence periods of common arable weed species in Rostock and Dijon. Emergence periods start earlier in spring and end later in autumn in Dijon. The same phenomenon occurs between Burgundy and Sweden: germination patterns are shifted by one month [29]. While for species in more Northern locations it would be the priority to avoid cold and frost injury to their sensitive seedlings (thus explaining spring germination at higher temperatures), drought and hot temperatures will pose the highest risk for seedling establishment in more Southern habitats [62] (explaining also the later start of fall emergence).

Figure 5: Main emergence periods of common arable weed species compared to field times of typical crops in North Eastern Germany and Burgundy. (Expert knowledge [14], Pulkenat pers. comm.)

Of the two functions of base temperature, the spring part may be more important for population fitness than the autumn part. Most weed species display pronounced emergence flushes. In autumn, the flush will start as soon as moisture is available. When temperatures decrease down to the base temperature, probably most non-dormant seeds will have already germinated and grown to a stage tolerant to frost. In spring on the other hand, the flush may start as soon as temperatures rise, with many seeds germinating at a time when temperatures may still quickly fall below zero, potentially leading to high losses due to frost. In spring, a potential loss of very young seedlings would therefore be much more dramatic in numbers than in autumn.

We can find more evidence for this mechanism when looking at species that are facultative autumn and spring germinators or strictly limited to one of the seasons, especially when this habit differs between sites. A shift in germination habit has been reported for a number of species [61]. *G. dissectum* is an example species that only germinates in autumn in Dijon, but in Rostock also in spring. The shift in germination period may explain the large difference of base temperature for *G. dissectum* (4°C in Rostock vs. 0°C in Dijon) [63]. We suspect *G. dissectum* to be (mostly) dormant in spring in Dijon. The base temperature therefore has no function to prevent seeds from germinating too early in spring, and subsequently selection pressure may never have favoured adaptation towards a higher base temperature. Similar differences in germination timing between provenances have been reported for *C. bursa-pastoris* [64] and *C. canadensis* [60].

Interaction of climate and management controls germination in early and late summer

We propose that the main factors driving germination and emergence of arable weeds in spring and autumn months are weather and management and their interaction. Management is similarly dependent on favourable periods as various weed life cycle stages.

The summer germinating weed species are the only ones that emerge at the same time in Rostock and Dijon. At this time (end of April/beginning of May), soil temperatures become similar in Dijon and Rostock after a long spell of colder soils in Rostock (Figure 1). Emergence timing coincides with the last tillage and sowing operations for maize cultivation. In contrast to most other crops, maize sowing is also synchronous between the two locations (Figure 5). Base temperatures in summer germinators were always higher in Rostock, but it remains unclear which adaptation and selection processes happened, because there seems to be no function for base temperature in these species.

Disparities between location-specific emergence patterns can arise due to different soil and crop management practices [65] and the pattern eventually is memorized by genetic adaptation. The later start of tillage and cultivation for winter-sown crops in Dijon (optimizing crop management) leads to selection in the weed species to emerge later (after last tillage). A similar effect of management

timing on germination was found for *Datura stramonium* seeds from three Southern European populations [33].

Intra-specific variability in early growth rates is much smaller than inter-specific variability

Early relative growth rates for three of the four species measured in Rostock agree with literature values [21]. Only the growth rate for *V. hederifolia* was slower than in any other previously measured weed species. To our knowledge, this study is the first to investigate inter-population differences in early growth rates of (non-woody) plant species, apart from [31]. Other studies on early growth rate often measured above-ground biomass rather than leaf area [21,26,66–70], which makes it hard to compare ranges.

Our results on relative growth rate were most strongly determined by species identity. This high inter-specific variability is of course related to the original species choice in Dijon that aimed to include a range of contrasting species into the experiments. We found a significant population effect on relative growth rate only for one species. This result is in agreement with earlier studies which found no maternal effects on life cycle stages later than germination [56].

In general, the inter-population or intra-specific variability decreased when a species was tested more than once, probably by a combination of lowering the experimental error as well as exploring a wider range of growing conditions with each population leading to some overlap and convergence. For some species, we found a very high variability in growth rates between successive trial with the same species and population indicating a plastic response to varying environmental conditions.

Higher inter-specific variation compared to intra-specific variation in growth responses under these non- stressful conditions is in contrast to earlier evidence that within-species variation in growth responses can be as high as among-species variation, albeit under stressful conditions [71]. The differences may be attributed to only two locations having been tested here (vs. several locations in Europe in [71]) and more numerous species chosen to represent among-species variation (15 vs. 4 species). It is therefore important to consider the species distributions when choosing populations

for testing intra-specific variation. Nonetheless, growing conditions (with or without stress), species identity and population selection all influence the balance of inter- and intra-specific variation and should be considered in future studies.

Early relative growth rate measured in relation to thermal time may ignore sensitivity to light quality

For *A. myosuroides*, the high intra-specific variability in our data (Figure 3) might be caused by the differences in light availability during the experimental runs, which happened in different seasons of the year. The species is a strict autumn germinator in Germany, therefore the experiment in Rostock took place in autumn. In Dijon, the experiment took place in spring. The species is a facultative spring and autumn germinator there. It was reported from growth experiments in contrasting seasons that seedlings of the same species grown in autumn had a lower relative growth rate per degree-day than the ones that emerged in spring which was explained by the lower light availability [26].

The positive relationship between base temperature and relative growth rate was also apparent in another analysis of 49 species tested in Dijon [21]. This could be caused by two different resource-related phenomena: the trade-off between base temperature and rate of biological processes [55], and the benefit from increased light availability for plants growing in longer days with higher solar radiation [70]. Our finding that plants in consecutive months had higher RGRs (Fig 4 in Supporting information) also supports this.

Conclusion

We have shown that local adaptation can be strongly trait-specific, posing the question whether certain traits are under higher selection pressure or are simply more plastic. With respect to implications for future weed models, our results provide additional criteria for explicitly incorporating intra-specific trait variability within the part simulating germination. In the context of typical weed modelling research where the scale of study is often local to regional and centred on sites with a weed community rather than one single species, intra-specific trait variability should be

included if the focus is on response traits like the community assembly. It might be negligible if the focus is laid more on effect traits such as ecosystem functioning or net primary productivity.

We suggest to increase accuracy of future modelling exercises within a study region by using germination base temperatures from local seed populations. There seems to be no need to specifically measure early growth rates from local populations, although further experiments with new populations increase the precision of species trait averages and ranges.

Acknowledgements

The French seeds were provided by the INRAE weed seed collection (UMR Agroécologie, INRAE Dijon).

References

1. Zakharova L, Meyer KM, Seifan M. Trait-based modelling in ecology: A review of two decades of research. Ecological Modelling. 2019; 407: 108703. doi: 10.1016/j.ecolmodel.2019.05.008.
2. Bàrberi P, Bocci G, Carlesi S, Armengot L, Blanco-Moreno JM, Sans FX. Linking species traits to agroecosystem services: a functional analysis of weed communities. Weed Res. 2018; 58: 76–88. doi: 10.1111/wre.12283.
3. Storkey J, Holst N, Bøjer OQ, Bigongiali F, Bocci G, Colbach N, et al. Combining a weed traits database with a population dynamics model predicts shifts in weed communities. Weed Research. 2015; 55: 206–218. doi: 10.1111/wre.12126.
4. Shipley B, Bello F de, Cornelissen JHC, Laliberté E, Laughlin DC, Reich PB. Reinforcing loose foundation stones in trait-based plant ecology. Oecologia. 2016; 180: 923–931. doi: 10.1007/s00442-016-3549-x.
5. Siefert A, Violle C, Chalmandrier L, Albert CH, Taudiere A, Fajardo A, et al. A global meta‐analysis of the relative extent of intraspecific trait variation in plant communities. Ecology Letters. 2015; 18: 1406‐1419. doi: 10.1111/ele.12508.
6. Violle C, Enquist BJ, McGill BJ, Jiang L, Albert CH, Hulshof C, et al. The return of the variance: intraspecific variability in community ecology. Trends Ecol Evol (Amst). 2012; 27: 244–252. doi: 10.1016/j.tree.2011.11.014.
7. Albert CH, Grassein F, Schurr FM, Vieilledent G, Violle C. When and how should intraspecific variability be considered in trait-based plant ecology. Perspectives in Plant Ecology, Evolution and Systematics. 2011; 13: 217–225. doi: 10.1016/j.ppees.2011.04.003.

8. Vellend M, Lajoie G, Bourret A, Múrria C, Kembel SW, Garant D. Drawing ecological inferences from coincident patterns of population- and community-level biodiversity. Mol Ecol. 2014; 23: 2890–2901. doi: 10.1111/mec.12756.

9. Saatkamp A, Cochrane A, Commander L, Guja LK, Jimenez-Alfaro B, Larson J, et al. A research agenda for seed-trait functional ecology. New Phytol. 2019; 221: 1764–1775. doi: 10.1111/nph.15502.

10. Violle C, Navas M-L, Vile D, Kazakou E, Fortunel C, Hummel I, et al. Let the concept of trait be functional! Oikos. 2007; 116: 882–892. doi: 10.1111/j.0030-1299.2007.15559.x.

11. Hodgson JG, Wilson PJ, Hunt R, Grime JP, Thompson K. Allocating C-S-R plant functional types: a soft approach to a hard problem. Oikos. 1999; 85: 282–294.

12. Weiher E, van der Werf A, Thompson K, Roderick M, Garnier E, Eriksson O. Challenging Theophrastus: A common core list of plant traits for functional ecology. Journal of Vegetation Science. 1999; 10: 609–620.

13. Garnier E, Navas ML. A trait-based approach to comparative functional plant ecology. Concepts, methods and applications for agroecology. A review. Agronomy for Sustainable Development. 2012; 32: 365–399. doi: 10.1007/s13593-011-0036-y.

14. Gardarin A, Guillemin J-P, Munier-Jolain NM, Colbach N. Estimation of key parameters for weed population dynamics models. Base temperature and base water potential for germination. European Journal of Agronomy. 2010; 32: 162–168. doi: 10.1016/j.eja.2009.09.006.

15. Gardarin A, Dürr C, Colbach N. Modeling the dynamics and emergence of a multispecies weed seed bank with species traits. Ecological Modelling. 2012; 240: 123–138. doi: 10.1016/j.ecolmodel.2012.05.004.

16. Bourgeois B, Munoz F, Fried G, Mahaut L, Armengot L, Denelle P, et al. What makes a weed a weed? A large-scale evaluation of arable weeds through a functional lens. American Journal of Botany. 2019; 106: 90–100. doi: 10.1002/ajb2.1213.

17. Grime JP. Evidence for the existence of three primary strategies in plants and its relevance to ecological and evolutionary theory. The American Naturalist. 1977; 111: 1169–1194.

18. Bischoff A, Vonlanthen B, Steinger T, Müller-Schärer H. Seed provenance matters — Effects on germination of four plant species used for ecological restoration. Basic and Applied Ecology. 2006; 7: 347–359. doi: 10.1016/j.baae.2005.07.009.

19. Jana S, Upadhyaya MK, Acharya SN. Genetic basis of dormancy and differential response to sodium azide in Avena fatua seeds. Canadian Journal of Botany. 1988; 66: 635–641.

20. Zhang H, Ransom C, Ludwig P, van Nocker S. Genetic analysis of early flowering mutants in arabidopsis defines a class of pleiotropic developmental regulator required for expression of the flowering-time switch flowering locus C. Genetics. 2003; 164: 347–358.

21. Colbach N, Moreau D, Dugué F, Gardarin A, Strbik F, Munier-Jolain N. The response of weed and crop species to shading. How to predict their morphology and plasticity from species traits and ecological indexes. European Journal of Agronomy. in rev.

22. Gaba S, Perronne R, Fried G, Gardarin A, Bretagnolle F, Biju-Duval L, et al. Response and effect traits of arable weeds in agro-ecosystems. A review of current knowledge. Weed Res. 2017; 57: 123–147. doi: 10.1111/wre.12245.

23. Borgy B, Perronne R, Kohler C, Grison A-L, Amiaud B, Gaba S. Changes in functional diversity and intraspecific trait variability of weeds in response to crop sequences and climate. Weed Res. 2016; 56: 102–113. doi: 10.1111/wre.12190.

24. Perronne R, Gaba S, Cadet E, Le Corre V. The interspecific and intraspecific variation of functional traits in weeds: diversified ecological strategies within arable fields. Acta Botanica Gallica. 2014; 161: 243–252. doi: 10.1080/12538078.2013.868320.

25. Gardarin A, Dürr C, Colbach N. Which model species for weed seedbank and emergence studies? A review. Weed Research. 2009; 49: 117–130. doi: 10.1111/j.1365-3180.2008.00683.x.

26. Storkey J. Modelling seedling growth rates of 18 temperate arable weed species as a function of the environment and plant traits. Annals of Botany. 2004; 93: 681–689. doi: 10.1093/aob/mch095.

27. Cochrane A, Yates CJ, Hoyle GL, Nicotra AB. Will among - population variation in seed traits improve the chance of species persistence under climate change. Global Ecology and Biogeography. 2015; 24: 12 - 24. doi: 10.1111/geb.12234.

28. Loddo D, Sousa E, Masin R, Calha I, Zanin G, Fernández-Quintanilla C, et al. Estimation and Comparison of Base Temperatures for Germination of European Populations of Velvetleaf (Abutilon theophrasti) and Jimsonweed (Datura stramonium). Weed sci. 2013; 61: 443–451. doi: 10.1614/WS-D-12-00162.1.

29. Guillemin J-P, Bellanger S, Reibeil C, Darmency H. Longevity, dormancy and germination of Cyanus segetum. Weed Res. 2017; 57: 361–371. doi: 10.1111/wre.12267.

30. Karlsson LM, Milberg P. Variation within species and inter-species comparison of seed dormancy and germination of four annual Lamium species. Flora: Morphology, Distribution, Functional Ecology of Plants. 2008; 203: 409–420. doi: 10.1016/j.flora.2007.08.001.

31. Royo-Esnal A, Torra J, Conesa JA, Recasens J. Emergence and early growth of Galium aparine and Galium spurium. Weed Research. 2012; 52: 458–466. doi: 10.1111/j.1365-3180.2012.00939.x.

32. Colbach N, Chauvel B, Dürr C, Richard G. Effect of environmental conditions on Alopecurus myosuroides germination. I. Effect of temperature and light. Weed Res. 2002; 42: 210–221. doi: 10.1046/j.0043-1737.2002.00279.x.

33. Loddo D, Sousa E, Masin R, Calha IM, Zanin G, Fernández-Quintanilla C, et al. Germination response of local Southern European populations of Datura stramonium at a range of constant temperatures. Weed Res. 2014; 54: 356–365. doi: 10.1111/wre.12083.

34. Masin R, Loddo D, Benvenuti S, Zuin MC, Macchia M, Zanin G. Temperature and Water Potential as Parameters for Modeling Weed Emergence in Central-Northern Italy. Weed sci. 2010; 58: 216–222. doi: 10.1614/WS-D-09-00066.1.

35. Guillemin J-P, Gardarin A, Granger S, Reibel C, Munier-Jolain N, Colbach N. Assessing potential germination period of weeds with base temperatures and base water potentials. Weed Research. 2013; 53: 76–87. doi: 10.1111/wre.12000.

36. Ritz C, Pipper CB, Streibig JC. Analysis of germination data from agricultural experiments. European Journal of Agronomy. 2013; 45: 1–6. doi: 10.1016/j.eja.2012.10.003.

37. Arnold CY. The determination and significance of the base temperature in a linear heat unit system. Proceedings of the American Society for Horticultural Science. 1959; 74: 430–445.

38. Easlon HM, Bloom AJ. Easy Leaf Area: Automated digital image analysis for raopid and accurate measurement of leaf area. Applications in Plant Sciences. 2014; 2.

39. Trnka M, Olesen JE, Kersebaum KC, Skielvag AO, Eitzinger J, SEGUIN B, et al. Agroclimatic conditions in Europe under climate change. Global Change Biology. 2011; 17: 2298–2318. doi: 10.1111/j.1365-2486.2011.02396.x.

40. Onofri A, Carbonell EA, Piepho H-P, Mortimer AM, Cousens RD. Current statistical issues in Weed Research. Weed Res. 2010; 50: 5–24. doi: 10.1111/j.1365-3180.2009.00758.x.

41. R Core Team. R: A language and environment for statistical computing. Vienna, Austria: R Foundation for Statistical Computing; 2017.

42. Ritz C, Baty F, Streibig JC, Gerhard D. Dose-Response Analysis Using R. PLoS ONE. 2015; 10: e0146021. doi: 10.1371/journal.pone.0146021.

43. Bates D, Maechler M, Bolker B, Walker S. Fitting Linear Mixed-Effects Models Using lme4. Journal of Statistical Software. 2015; 67: 1–48.

44. Kuznetsova A, Brockhoff PB, Christensen RHB. lmerTest Package: Tests in Linear Mixed Effects Models. Journal of Statistical Software. 2017; 82: 1–26.

45. Lenth R. emmeans. Estimated Marginal Means, aka Least-Squares Means.; 2019.

46. Benvenuti S, Macchia M. Calculation of threshold temperature for the development of various weeds. Agr. Med. 1993; 123: 252–256.

47. Loddo D, Ghaderi-Far F, Rastegar Z, Masin R. Base temperatures for germination of selected weed species in Iran. Plant Protect Sci. 2017; 54: 60–66. doi: 10.17221/92/2016-PPS.

48. Gardarin A, Dürr C, Colbach N. Prediction of germination rates of weed species. Relationships between germination speed parameters and species traits. Ecological Modelling. 2011; 222: 626–636. doi: 10.1016/j.ecolmodel.2010.10.005.

49. Steinmaus SJ, Prather TS, Holt JS. Estimation of base temperatures for nine weed species. Journal of Experimental Botany. 2000; 51: 275–286. doi: 10.1093/jexbot/51.343.275.

50. Izquierdo J, González-Andújar JL, Bastida F, Lezaún JA, del Arco MJS. A Thermal Time Model to Predict Corn Poppy (Papaver rhoeas) Emergence in Cereal Fields. Weed sci. 2009; 57: 660–664. doi: 10.1614/WS-09-043.1.

51. Masin R, Zuin MC, Archer DW, Forcella F, Zanin G. WeedTurf: a predictive model to aid control of annual summer weeds in turf. Weed Sci. 2005; 53: 193–201. doi: 10.1614/WS-04-066R1.

52. Grundy AC, Phelps K, Reader RJ, Burston S. Modelling the germination of Stellaria media using the concept of hydrothermal time. New Phytologist. 2000; 148: 433–444. doi: 10.1046/j.1469-8137.2000.00778.x.

53. Thompson PA. Characterization of the Germination Responses of Silene dioica (L.) Clairv., Populations from Europe. Annals of Botany. 1975; 39: 1–19. doi: 10.1093/oxfordjournals.aob.a084909.

54. Rosbakh S, Poschlod P. Initial temperature of seed germination as related to species occurrence along a temperature gradient. Funct Ecol. 2015; 29: 5–14. doi: 10.1111/1365-2435.12304.

55. Trudgill DL, Honek A, LI D, van Straalen NM. Thermal time – concepts and utility. Annals of Applied Biology. 2005; 146: 1–14. doi: 10.1111/j.1744-7348.2005.04088.x.

56. Bischoff A, Müller ‐ Schärer H. Testing population differentiation in plant species – how important are environmental maternal effects. Oikos. 2010; 119: 445 – 454. doi: 10.1111/j.1600-0706.2009.17776.x.

57. Baskin CC, Milberg P, Andersson L, Baskin JM. Germination ecology of seeds of the annual weeds Capsella bursa-pastoris and Descurainia sophia originating from high northern latitudes. Weed Res. 2004; 44: 60–68. doi: 10.1046/j.1365-3180.2003.00373.x.

58. Williams JT, Harper.J.L. Seed polymorphism and germination I. The influence of nitrate and low temperatures on the germination of Chenopodium album. Weed Research. 1965; 5: 141–150.

59. van der Vegte FW. Population differentiation and germination ecology in Stellaria media (L.) Vill. Oecologia. 1978; 37: 231–245. doi: 10.1007/BF00344994.

60. Tozzi E, Beckie H, Weiss R, Gonzalez-Andujar JL, Storkey J, Cici SZH, et al. Seed germination response to temperature for a range of international populations of Conyza canadensis. Weed Res. 2014; 54: 178–185. doi: 10.1111/wre.12065.

61. Cici SZ, Van Acker RC. A review of the recruitment biology of winter annual weeds in Canada. Canadian Journal of Plant Science. 2009; 89: 575–589. doi: 10.4141/CJPS08131.

62. Fenner M, Thompson K, editors. The ecology of seeds. Cambridge: Cambridge University Press; 2005.

63. Gardarin A, Colbach N. How much of seed dormancy in weeds can be related to seed traits. Weed Res. 2015; 55: 14–25. doi: 10.1111/wre.12121.

64. Baskin JM, Baskin CC. Germination responses of buried seeds of Capsella bursa ‐ pastoris exposed to seasonal temperature changes. Weed Res. 1989; 29: 205 – 212. doi: 10.1111/j.1365-3180.1989.tb00860.x.

65. Forcella F, Benech Arnold RL, Sanchez R, Ghersa CM. Modeling seedling emergence. Field Crops Research. 2000; 67: 123–139. doi: 10.1016/S0378-4290(00)00088-5.

66. Fayaud B, Coste F, Corre-Hellou G, Gardarin A, Dürr C. Modelling early growth under different sowing conditions: A tool to predict variations in intercrop early stages. European Journal of Agronomy. 2014; 52: 180–190. doi: 10.1016/j.eja.2013.09.009.

67. Fenner M. Relationships between seed weight, ash content and seedling growth in twenty-four species of Compositae. New Phytologist. 1983; 95: 697–706.

68. Prieto I, Litrico I, Violle C, Barre P. Five species, many genotypes, broad phenotypic diversity: When agronomy meets functional ecology. American Journal of Botany. 2017; 104: 62–71. doi: 10.3732/ajb.1600354.

69. Storkey J, Cussans JW. Relationship between temperature and the early growth of Triticum aestivum and three weed species. Weed Science. 2000; 48: 467–473. doi: 10.1614/0043-1745(2000)048[0467:RBTATE]2.0.CO;2.

70. Hunt R, Cornelissen JHC. Components of relative growth rate and their interrelations in 59 temperate plant species. New Phytol. 1997; 135: 395–417. doi: 10.1046/j.1469-8137.1997.00671.x.

71. Malyshev AV, Arfin Khan MAS, Beierkuhnlein C, Steinbauer MJ, Henry HAL, Jentsch A, et al. Plant responses to climatic extremes: within-species variation equals among-species variation. Global Change Biology. 2016; 22: 449–464. doi: 10.1111/gcb.13114.

Supporting information

S1 File. Supporting Information

Figure1

Click here to access/download;Figure;Fig1.tif

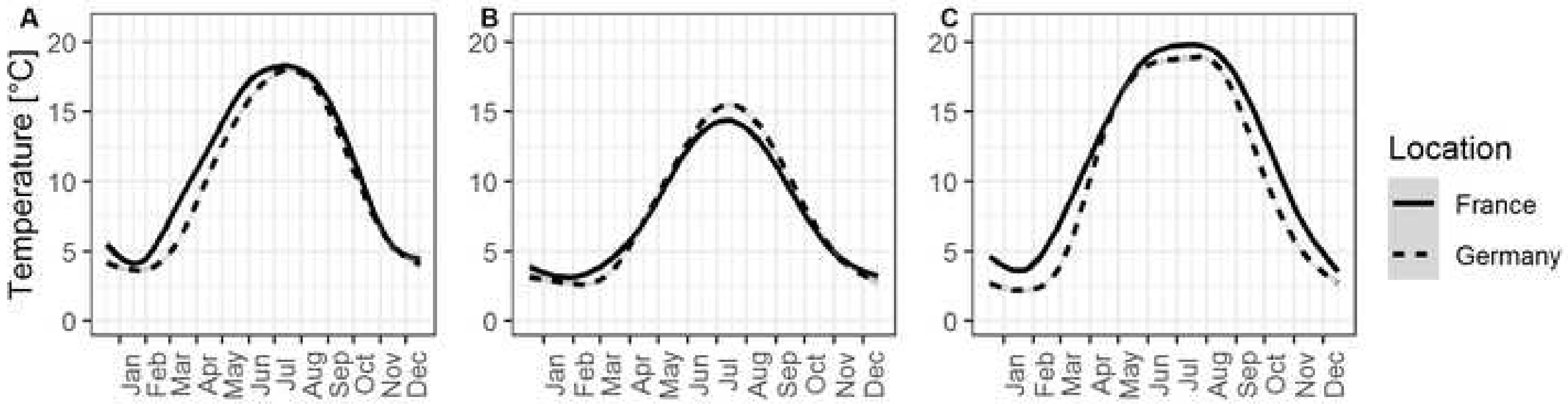

Figure2

Click here to access/download;Figure;Fig2.tif

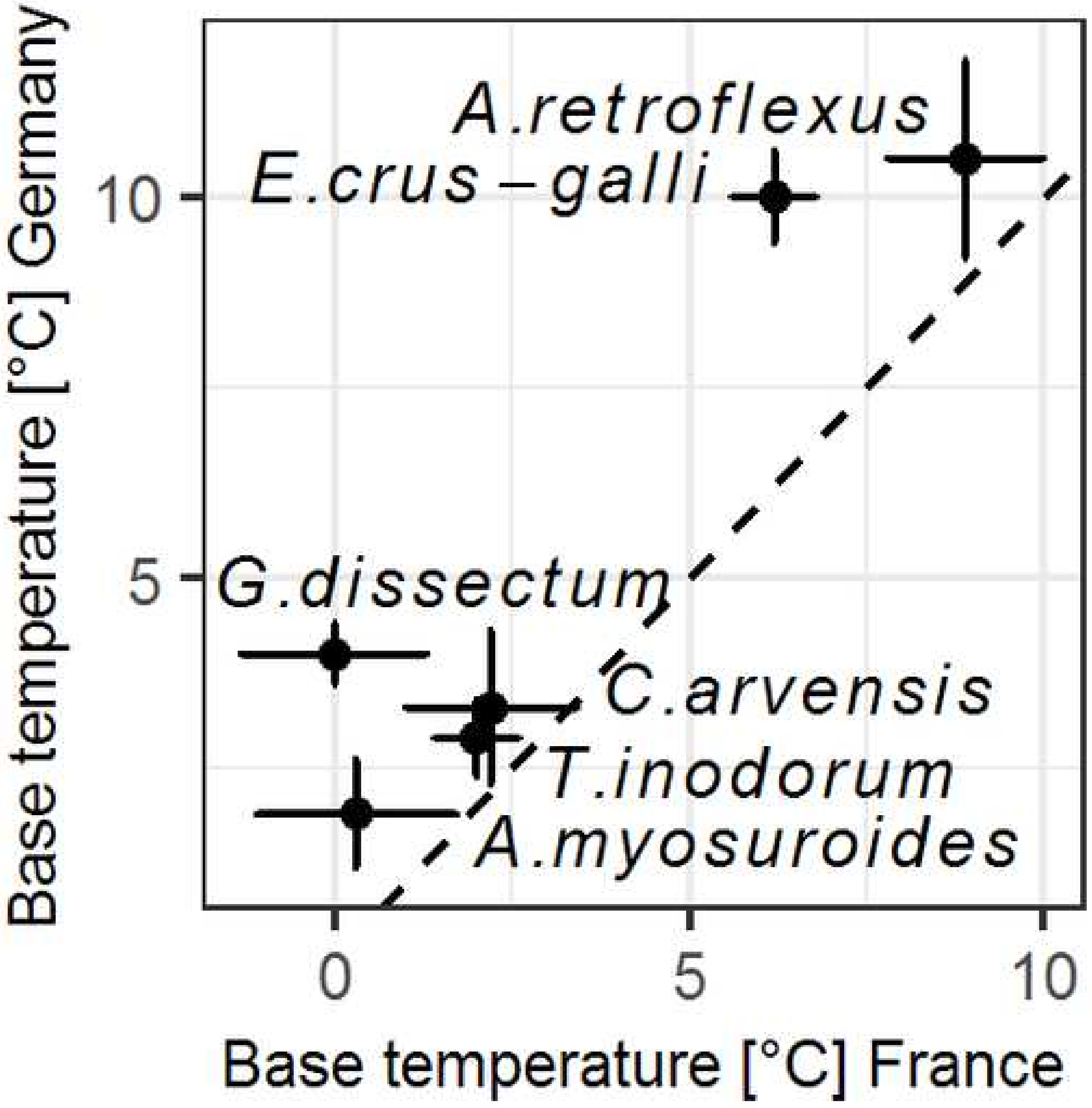

Figure3

Click here to access/download;Figure;Fig3.tif

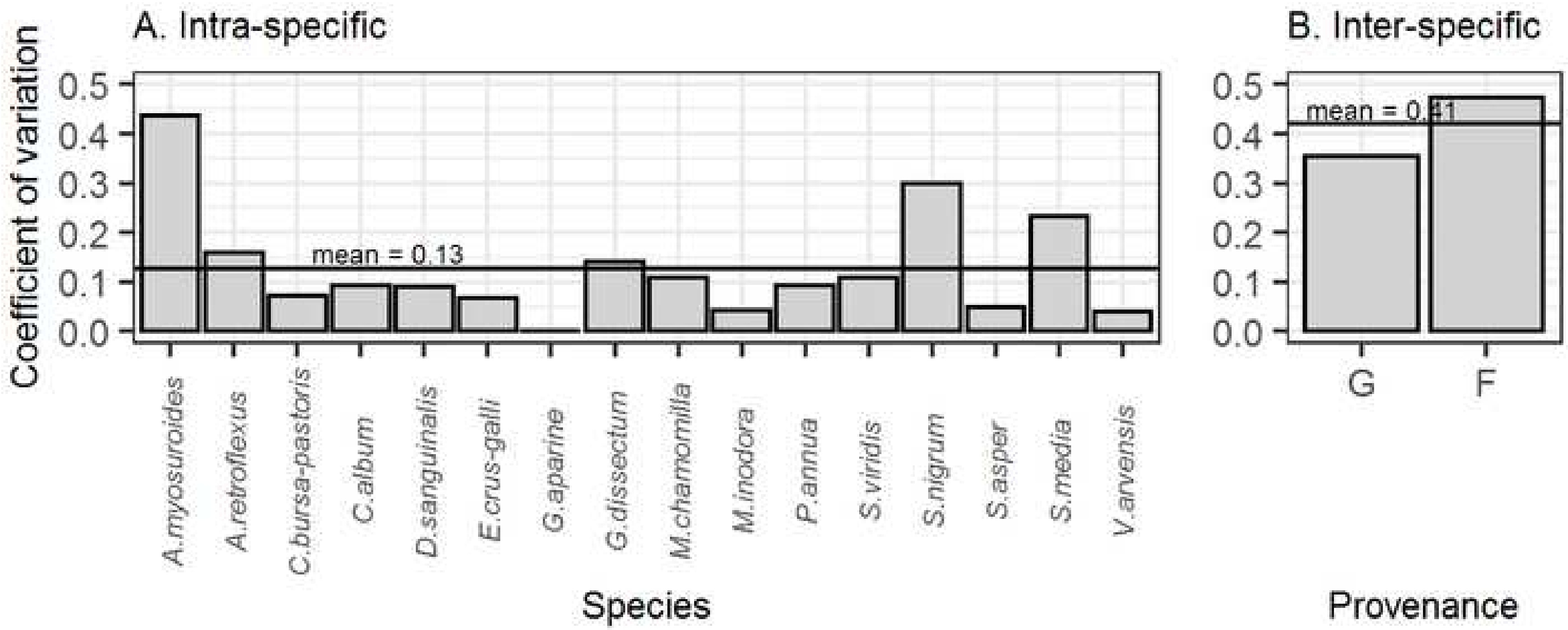

Figure4

Click here to access/download;Figure;Fig4.tif

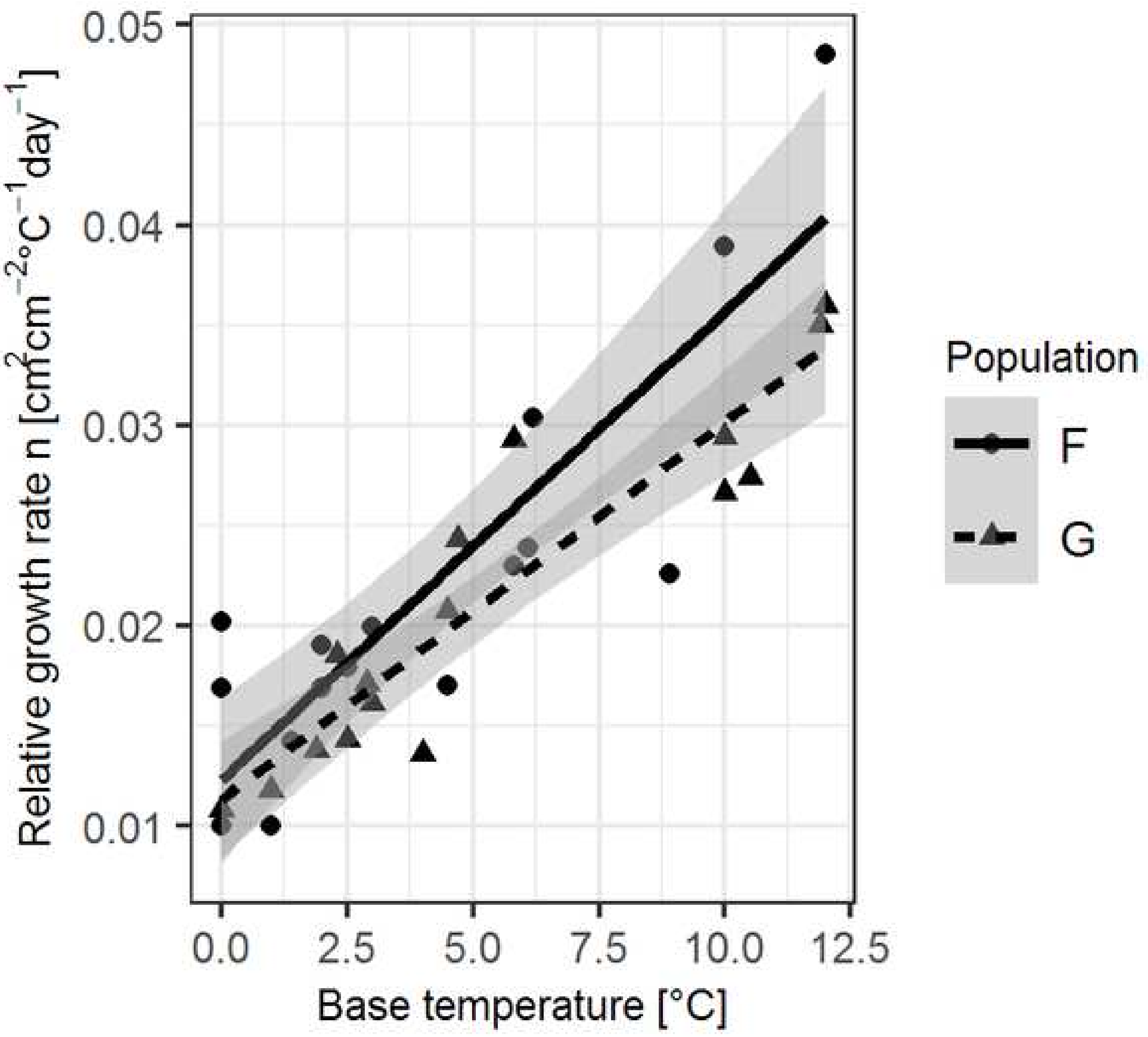

Figure5

Click here to access/download;Figure;Fig5.tif

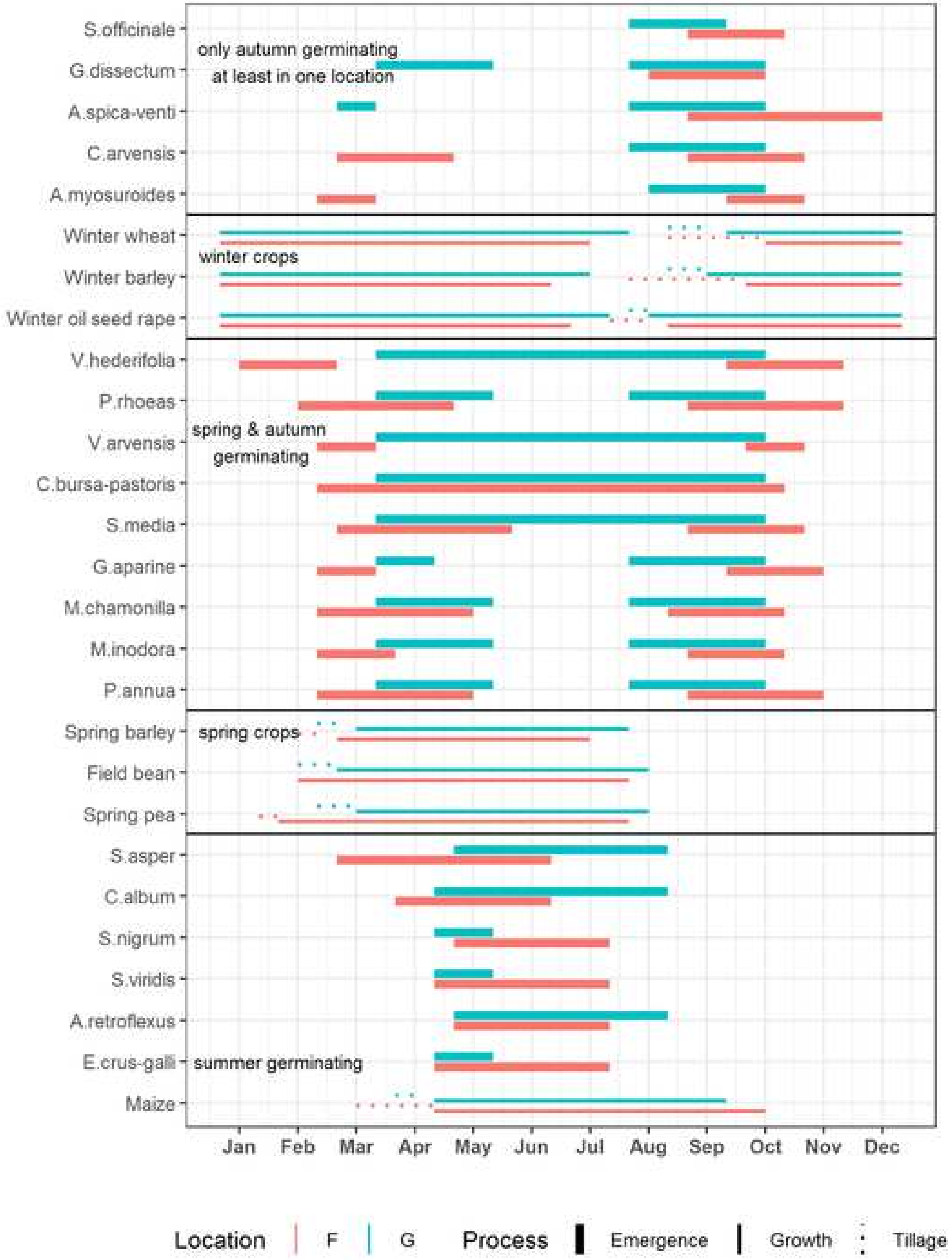

Click here to access/download
Supporting Information
Supporting Information.docx

Data Review URL

Click here to download Data Review URL
https://datadryad.org/stash/share/d57vxvQFtZ45n6_MQyrMOAG7So6T0
